# Juvenile-Onset Recurrent Respiratory Papillomatosis Aggressiveness: In Situ Study of the Level of Transcription of HPV E6 and E7

**DOI:** 10.3390/cancers12102836

**Published:** 2020-10-01

**Authors:** Charles Lépine, Thibault Voron, Dominique Berrebi, Marion Mandavit, Marine Nervo, Sophie Outh-Gauer, Hélène Péré, Louis Tournier, Natacha Teissier, Eric Tartour, Nicolas Leboulanger, Louise Galmiche, Cécile Badoual

**Affiliations:** 1 Department of Pathology, European Hospital Georges-Pompidou, Assistance Publique-Hôpitaux de Paris, F-75015 Paris, France; charles.lepine@aphp.fr (C.L.); marine.nervo@gmail.com (M.N.); sophieouthgauer@gmail.com (S.O.-G.); 2 Université de Paris, PARCC, INSERM-U970, F-75015 Paris, France; thibault.voron@gmail.com (T.V.); marion.mandavit@gmail.com (M.M.); helene.pere@aphp.fr (H.P.); eric.tartour@aphp.fr (E.T.); 3 Department of Pathology, Robert Debré Hospital, Assistance Publique-Hôpitaux de Paris, F-75019 Paris, France; dominique.berrebi2@aphp.fr (D.B.); louis_tournier@yahoo.fr (L.T.); 4 Department of Virology, European Hospital Georges-Pompidou, Assistance Publique-Hôpitaux de Paris, F-75015 Paris, France; 5 Department of Pediatric ENT Surgery, Robert Debré Hospital, Assistance Publique-Hôpitaux de Paris, F-75019 Paris, France; natacha.teissier@aphp.fr; 6 Department of Immunology, European Hospital Georges-Pompidou, Assistance Publique-Hôpitaux de Paris, F-75015 Paris, France; 7 Department of Pediatric ENT Surgery, Necker-Enfants Malades Hospital, Assistance Publique-Hôpitaux de Paris, F-75015 Paris, France; nicolas.leboulanger@aphp.fr; 8 Department of Pathology, Necker-Enfants Malades Hospital, Assistance Publique-Hôpitaux de Paris, F-75015 Paris, France; louise.galmiche@gmail.com

**Keywords:** Juvenile-onset recurrent respiratory papillomatosis, HPV 6, HPV 11, RNA, chromogenic in situ hybridization

## Abstract

**Simple Summary:**

Juvenile-onset recurrent respiratory papillomatosis (JoRRP) is a condition related to HPV 6 and 11 infection which is characterized by the repeated growth of benign exophytic papilloma in the respiratory tract of children. Disease progression is unpredictable leading sometimes to airway compromise and death. The aim of this study was to explore p16^INK4a^ and expression of the RNA of HPV genes *E6* and *E7* with a chromogenic in situ hybridization (CISH) as biomarkers of JoRRP aggressiveness on a bicentric cohort of forty-eight children. CISH was scored semi-quantitatively as high (2+ score) and low (1+ score) levels of transcription of *E6* and *E7*. Patients with a 2+ score had a more aggressive disease compared to those with a 1+ score. These data are a first step towards the use of biomarkers predictive of disease severity in JoRRP, this could improve the disease management, for example, by implementing adjuvant treatment at the early stages.

**Abstract:**

Juvenile-onset recurrent respiratory papillomatosis (JoRRP) is a condition related to HPV 6 and 11 infection which is characterized by the repeated growth of benign exophytic papilloma in the respiratory tract. Disease progression is unpredictable: some children experience minor symptoms, while others require multiple interventions due to florid growth. The aim of this study was to explore the biomarkers of JoRRP severity on a bicentric cohort of forty-eight children. We performed a CISH on the most recent sample of papilloma with a probe targeting the mRNA of the *E6* and *E7* genes of HPV 6 and 11 and an immunostaining with p16^INK4a^ antibody. For each patient HPV RNA CISH staining was assessed semi-quantitatively to define two scores: 1+, defined as a low staining extent, and 2+, defined as a high staining extent. This series contained 19 patients with a score of 1+ and 29 with a score of 2+. Patients with a score of 2+ had a median of surgical excision (SE) per year that was twice that of patients with a score of 1+ (respectively 6.1 versus 2.8, *p* = 0.036). We found similar results with the median number of SE the first year. Regarding p16^INK4a^, all patients were negative. To conclude, HPV RNA CISH might be a biomarker which is predictive of disease aggressiveness in JoRRP, and might help in patient care management.

## 1. Introduction

Recurrent respiratory papillomatosis (RRP) is characterized by the repeated growth of benign exophytic papilloma in the respiratory tract [1,2], primarily in the larynx [1]. The age distribution of RRP is trimodal, with a first peak in children younger than 5 years of age, a second one in adults between 20 and 40 years old and a third in individuals around the age of 64 [3,4]. This condition is referred to as Juvenile-onset Recurrent respiratory papillomatosis (JoRRP) when it occurs in children. There is a limited number of studies with epidemiologic data: in Denmark, between 1969 and 1984 the incidence was 3.6 case per year per 100,000 children [5], whereas in Canada, based on a national database, the incidences and prevalences from 1994 to 2007 were, respectively, 0.24 and 1.11 per 100,000 children, and the median age at diagnosis was 4.4 years with a sex ratio near 1:1 [6]. In the United States of America, data are similar [7]; however, incidence and prevalence seems to depend on socioeconomic status [8]. JoRRP is caused by an HPV infection, mostly by 6 and 11 genotypes [9]. Three modes of transmission are suggested: vertical transmission at birth (however, HPV type concordance between mother and newborn in different studies are contradictory [10,11,12]), vertical transmission in utero [13] and horizontal transmission via the child’s environment [10]. Several studies have also demonstrated that maternal condyloma at the time of delivery is a major risk factor of developing JoRRP [14,15]. While the prevalence of HPV 6 and 11 infection in pregnant women is around 2%, the prevalence of JoRRP is surprisingly low. Thus, HPV infection alone does not explain the development of the disease, and strong arguments suggests that JoRRP is linked to immunity defects and genetic susceptibility. Patients with RRP are associated with HLA DRB1*0102/0301, DQB1*0201/0202 [16,17] and presents a lack of KIR genes 3DS1 et 2DS1 [18]. Moreover, their immune response presents a Th2 polarization [19,20] which is not suitable for viral infection control. 

The management of this disease is challenging, due to its unpredictable course: some children experience minor symptoms with spontaneous remission, while others require multiple interventions due to florid growth. In addition, RRP may lead to airway compromise. Malignant transformation to carcinoma rarely occurs, most often over pulmonary spread [21,22]. The standard treatment of JoRRP is a surgical excision (SE) with cold instruments or microdebriders. Multiple endolaryngeal procedures can lead to glottis synechia and irreversible damage to the vocal cords, as well as an impaired social life [23]. To improve the surgical outcome and extend the symptom-free interval, numerous adjuvant treatments have been tried: interferon α [24], celecoxib [25], bevacizumab [26], cidofovir [27,28], PD-1/PD-L1 immunotherapy [29,30] and the quadrivalent HPV vaccine [31]. Currently, routine use of these treatments is not recommended by the International Pediatric Otolaryngology Group [32]. The most promising approaches are the quadrivalent HPV vaccine, bevacizumab and PD-1/PD-L1 immunotherapies which appear to decrease relapses [29,30,33,34]. Systemic bevacizumab, a monoclonal antibody against VEGF-A, seems to be the most promising treatment for aggressive forms of JoRRP, as several studies found a rapid and sustained partial or complete response to treatment in patients with lung involvement [35,36]. In order to improve the management of these children with JoRRP, it seemed important to us to identify biomarkers associated with the severity of the disease. Many studies have focused on finding clinical severity risk factors, of which only early age of onset of the disease [37,38,39] is currently recognized as such. 

We previously described in p16 positive squamous cell carcinoma of the oropharynx two prognostic groups thanks to HPV RNA chromogenic in situ hybridization (CISH) [40]. In brief, HPV RNA CISH was scored semi-quantitatively as “high” and “low” depending on the extent of the staining. RNA CISH high staining was associated with a better overall survival in both univariate and multivariate analyses (*p* = 0.033 and *p* = 0.042, respectively). Based on these results, we decided to explore HPV E6 and E7 transcription with this technique in our cohort of JoRRP. Thus, we hypothesize that a highly transcriptionally active virus could be associated with more severe disease. When it comes to HPV related cancers, the p16 protein is typically essential. It is a surrogate marker of HPV related cancers in various locations, including oropharyngeal squamous cell carcinoma (OPSCC) [2]. p16 is a CDK (cyclin-dependent kinase) inhibitor. This protein is involved in the pRB pathway, implicated in cell cycle regulation; its overexpression avoids phosphorylation of Rb family members, leading to cell cycle arrest into G1 phase [41]. Low-risk HPV produce E6 and E7 proteins which have lower affinity for p53 and pRb proteins [42], and thus, are not theoretically associated with cell cycle progression, nor with p16 overexpression. Conversely, in lesions related with high risk HPV (cancers or intra-epithelial neoplasia), there is an overexpression of the p16 protein, resulting in intense cytoplasmic and nuclear staining of the majority of tumors cells (>70%); this has mainly been studied in the female genital tract and the anus [43,44,45]. However, low-risk HPV-related cancers have been described in different locations including the anus and head and neck [46,47,48]. Data over p16 expression are heterogeneous, with some studies finding p16 positivity in these cancers while others do not. 

The aim of this study is to explore p16 expression and HPV RNA CISH as in situ biomarkers of JoRRP severity.

## 2. Results

### 2.1. Population

Forty-eight children were included: twenty-two were boys and twenty-six were girls. The average age at diagnosis was 3.8 years, with a median of 2 years. Twenty-seven percent of patients had HPV 11 infection, 65% had HPV 6 infection and 6% had co-infection with HPV 6 and 11. All patients had glottic involvement; 73% of patients had supraglottic complication, 68.7% had subglottic localization, 25% had tracheal involvement and 8% had pulmonary lesions. Regarding adjuvant treatment, 73% of patients received at least one injection of Cidofovir. Patients received an average of 7.1 injections of Cidofovir with a median of 3.5 injections. Six patients (12.5%) received Cidofovir during an SE prior to the study specimen. The delay between the first and last SE was on average 3.6 years and the median was 2 years. Moreover, 71% of patients had a lesion at the last check-up. In addition, in our cohort, a young patient died at the age of 18 from the malignant transformation of a pulmonary localization of her JoRRP into bronchopulmonary squamous cell carcinoma. Her JoRRP progressed for 17 years: 132 SE were performed, with a mean interval between each endoscopy of 47 days. She also received 67 injections of Cidofovir.

### 2.2. p16 Immunochemistry

All patients had a negative staining with p16^INK4a^ antibody.

### 2.3. HPV RNA Chromogenic In Situ Hybridization

All negative control DaPB probes were negatives. For the PPIB housekeeping gene control probe, we found 11 patients with a score of 1+ and 37 with a score of 2+. No statistical correlation was found between the grading of the HPV E6 and E7 probe and the PPIB probe (*p* = 0,063, Chi2 test).

For the HPV RNA probe, we found 19 patients with a score of 1+ and 29 with a score of 2+. The characteristics of these two groups are described in Table 1. The two populations are comparable in terms of HPV type, gender and location of the lesions. The only patient who died of her disease had a score of 2+. Although patients with a score of 2+ had more lung involvement and tracheostomy, the difference was not statistically significant. 

The clinical markers of JoRRP aggressiveness are described in Table 2. Patients with a score of 2+ had a median of SE per year that was two times higher than that of patients with a score of 1+. We found similar results with the median number of SE the first year. Also, for the score of 2+, we found 72% patient with more than 4 SE in one year compared to patients with score of 1+, who comprised only 37%. Although the difference is not statistically different, patient with a score of 2+ had a median of interval between each SE two time shorter compared with 1+ patients. We also performed a second statistical analysis excluding the patient who underwent 132 SE and received 67 injections of Cidofovir; the results were similar. We found that patients with a score of 2+ had a median of SE per year that was higher than patients with a score of 1+ (respectively 5.8 versus 2.8, *p* = 0.039), and patients with a score of 2+ also had a higher median number of SE the first year (respectively 5 versus 2, *p* = 0.019). There were no significant differences between the two groups for the other items.

## 3. Discussion

Juvenile recurrent respiratory papillomatosis is a rare disease. In order to improve the management of these patients, it is necessary to carry out studies to find new biomarkers that could predict disease severity. To our knowledge, our cohort of JoRRP is the largest ever studied in Europe. Our population had characteristics which were comparable to those described in national databases in the U.S. and Canada (covering respectively 603 and 243 children with JoRRP [6,49]). We found a median rate of SE per year of 4.8, comparable to the U.S. cohort, which was of 4.3, higher than the Canadian one, i.e., 1.5. Our median age at diagnosis was slightly lower, i.e., 2 years old versus 3 years old in the U.S. cohort and 4 years old in the Canadian one. Interestingly, the percentage of patients treated with Cidofovir was much higher in our cohort than in the Canadian cohort (respectively 73% vs. 4.7%). The differences in terms of Cidofovir treatment could be explained by variability in local practices. Regarding the distribution of HPV types, our data are comparable to those presented in the literature. We found a low proportion of co-infection with HPV6 and 11 (6%) and a predominance of HPV6 (65%), as described elsewhere [50,51]. Currently, no consensual definition exists in the literature for disease severity. Some authors use composite scores incorporating criteria for disease localization, such as the Derkay-Coltrera score, and intervention-related criteria, such as the number of SE per year [6,38,52]. Others use only intervention-related criteria. A total number of SE greater than or equal to 10 or a number of SE/year greater than 3 or 4 is frequently found as a criterion of severity [37]. We were unable to use Derkay-Coltrera score because one of the two centers involved did not use it systematically. Given the absence of consensus, we remained descriptive and compared known intervention-related criteria between our two groups of JoRRP. Thus, our results with the E6 and E7 RNA CISH can be correlated to the activity of the disease and, by inference, to its severity.

To date, E6 and E7 RNA CISH has not been tested in benign HPV-related tumors. It is a great opportunity to have performed, for the first time, a CISH with an HPV6/11 RNA probe on an JoRRP cohort. As expected, all the patients had a positive CISH for E6 and E7 RNA of HPV6/11, confirming the presence of viral transcription in the papilloma of JoRRP. Both populations were comparable in terms of HPV type, gender and location of the lesions. Our results show an association between patients with a score of 2+ and higher activity markers of the disease. Indeed, patients with score of 2+ had a median of SE per year and a median number of SE the first year, twice as high as those of patients with score of 1+; this difference is statistically significant. We also found that patient with a score of 2+ were associated with more than 4 SE in one year, compared to patient with score of 1+. The difference between the two groups for the mean interval between each SE was also important, but not statistically significant. Another striking result was the higher frequency of Cidofovir treatment and the higher number of injection of Cidofovir in patients with a score of 2+; however, these results were close to statistical significance. Another data pointing in this direction is that the patient who died from a carcinomatous transformation of her JoRRP had a score of 2+. Our statistical analyses were not influenced by this patient (who underwent 132 SE and received 67 injections of Cidofovir), since a second statistical analysis was performed excluding this patient, with very similar results. Considering these results, patients with a high level of E6 and E7 transcription (score of 2+) had a more aggressive disease compared those with a low level of transcription (score of 1+). The opposite was observed in our study with OPSCC, as patients with a score of 2+ had a better overall survival compared to those with a score of 1+ [40]. This is likely explained by the immune response failure context in JoRRP, while in OPSCC the presence of more viral RNA in tumors cells could boost the immune response. Our semi-quantitative results with this technique are reliable, since our negative control probes (DaPB) were all negative and our control probes of a housekeeping gene were all positive with a semi-quantitative score not being correlated to the HPV6/11 probe score. However, injection of Cidofovir prior to the SE is a possible confounding factor. Cidofovir is a nucleotide analogue that blocks the DNA replication of viruses by inhibiting their DNA polymerase [53]. The effect of Cidofovir on E6 and E7 mRNA expression is unknown, although it can theoretically be assumed that Cidofovir decreases viral transcription by limiting the number of replicated viral DNA molecules. Thus, it would be interesting to carry out a study comparing the CISH score on samples before and after Cidofovir injection and to look for a correlation with the response to treatment. It can be hypothesized that the HPV6/11 RNA probe could help predict the response to Cidofovir, and thus avoid giving injections to non-responders. The observation of the patient who died from a malignant transformation of her JoRRP seems to be a first argument for the possibility of predicting the response to Cidofovir. Indeed, this patient had received 67 Cidofovir injections, which did not control the disease; she had a CISH score of 2+, with the HPV6/11 RNA probe on the sample taken after receiving Cidofovir injections.

Regarding immunohistochemistry with the p16^INK4a^ antibody, we found, as expected, that there was no overexpression of p16 in the papilloma of JoRRP, even for the patient who died of a carcinomatous transformation. It should be noted, however, that the p16^INK4a^ antibody was performed on a squamous papilloma collected before the onset of the transformation. In addition, no sample of the transformation was available for this patient. Interestingly, Huebbers et al. described a case of transformation of JoRRP related to HPV6. They did not find a p16 positivity in the carcinomatous sample, or in the papilloma sample, but they revealed a single site integration of the viral DNA in the carcinoma sample, while for the papilloma sample, the viral DNA was episomal [54]. Our results are consistent with the data in the literature. Indeed, in a series of condyloma acuminata (mostly related to HPV6 and 11), none showed homogeneous and diffuse p16 staining [55].

## 4. Materials and Methods 

### 4.1. Population

This retrospective study was approved by an ethical committee (notice number: CPP2019-02’-019a/2019-00352-55/19.02.05.67237) and by the “Commission Nationale Informatique et Libertés” (application number: 919150). Patients were selected from two pediatric University Hospitals (CHU) in Paris that treat JoRRP: Necker-Enfants Malades Hospital and Robert Debré Hospital. These two hospitals had similar protocols to treat JoRRP, and a SE was performed when patients were dyspneic or when the lesion growth was exponential. One sample per patient was selected, i.e., the most recent one, offering the best possible RNA quality. The inclusion criteria were:

- at least one available sample of laryngeal squamous papilloma.
- for each patient at least one positive in situ hybridization with an HPV “low risk” DNA probe or positive PCR for HPV 6 and/or 11.
- at least one recurrence after diagnosis.

The clinical data were collected retrospectively in March 2018. The following was collected for each patient: gender, age at diagnosis, dates of each SE performed in the two University Hospitals, number of SE, number of Cidofovir injections received, tracheotomy in relation to the disease, presence of surgical sequelae defined as the appearance of synechia at the glottic stage or even stenosis, location of papillomas, presence of lung involvement proven by at least one chest CT scan, presence of a lesion at the last check-up nasofibroscopy, carcinomatous transformation of JoRRP lesions, death related to the disease and cidofovir injection dates to determine whether the sample studied was taken before or after Cidofovir treatment.

From the dates of the SE, an average interval in days between each SE was calculated. The number of SE per year was calculated by dividing the total number of SE by the time in years between the first and last SE.

### 4.2. HPV RNA Chromogenic In Situ Hybridization

We used RNAscope^®^ kits (Advanced Cell Diagnostics Inc., Newak, CA, USA) from the manufacturer ACD™ on FFPE sections of papilloma. For each patient, we used the most recent sample of papilloma (to avoid RNA degradation):

- An in situ hybridization with a probe targeting the mRNA of the E6 and E7 proteins of HPV 6 and 11.
- A negative control with an in situ hybridization probe targeting the RNA of the Bacillus subtilis dihydrodipicolinate reductase bacterial gene transcript (probe name: DaPB).
- A positive control with an in situ hybridization probe targeting the RNA of the Cyclophilin B housekeeping gene (probe name: PPIB), an ubiquitous housekeeping gene.

The FFPE sections were stored at 4 °C before the technique was carried out. The hybridizations were performed according to our laboratory protocol [56]. For each batch of CISH with the RNA probe of HPV 6 and 11, an external positive control was performed. 

**Figure 1:**
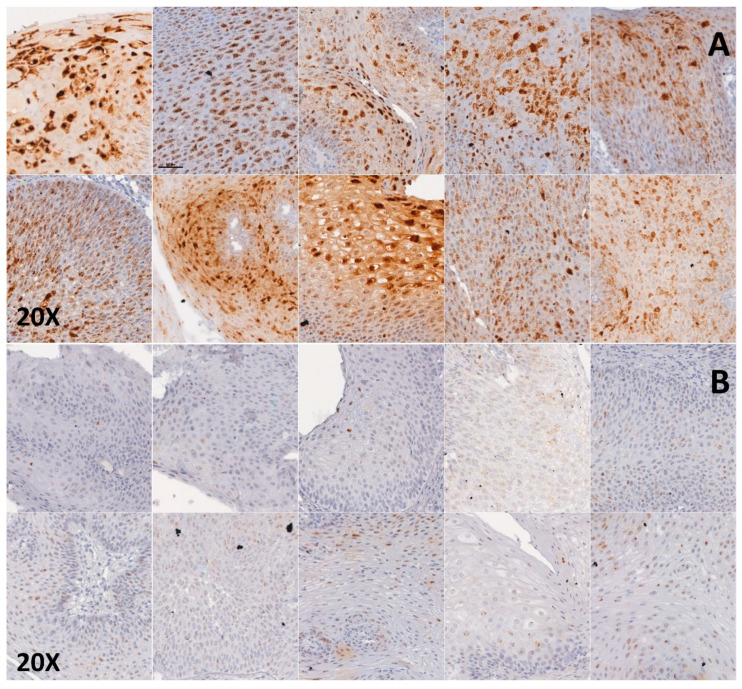
E6 and E7 HPV RNA CISH in papillomas of JoRRP: (**A**) 10 patients with score 2+: at x20 magnification, staining of more than 50% of the cells, or staining of more than 80% of the cell surface in at least 30% of the tumor cells. (**B**) 10 patients with score 1+: at x20 magnification, staining less than or equal to 50% of the cells, or staining of more than 80% of the cell surface in less than 30% of the tumor cells.

Each slide was semi-quantitatively scored by three pathologists together (C.B., C.L. and L.G), each slide was assigned a score of 0, 1+ or 2+, as described in Figure 1:

- Score 0: no staining
- Score 1+: at ×20 magnification, staining less than or equal to 50% of the cells, or staining of more than 80% of the cell surface in less than 30% of the tumor cells.
- Score 2+: at ×20 magnification, staining of more than 50% of the cells, or staining of more than 80% of the cell surface in at least 30% of the tumor cells.

Staining with PPIB probe was also assessed semi-quantitatively following the same rules as previously described.

### 4.3. p16 Immunochemistry

**Figure 2:**
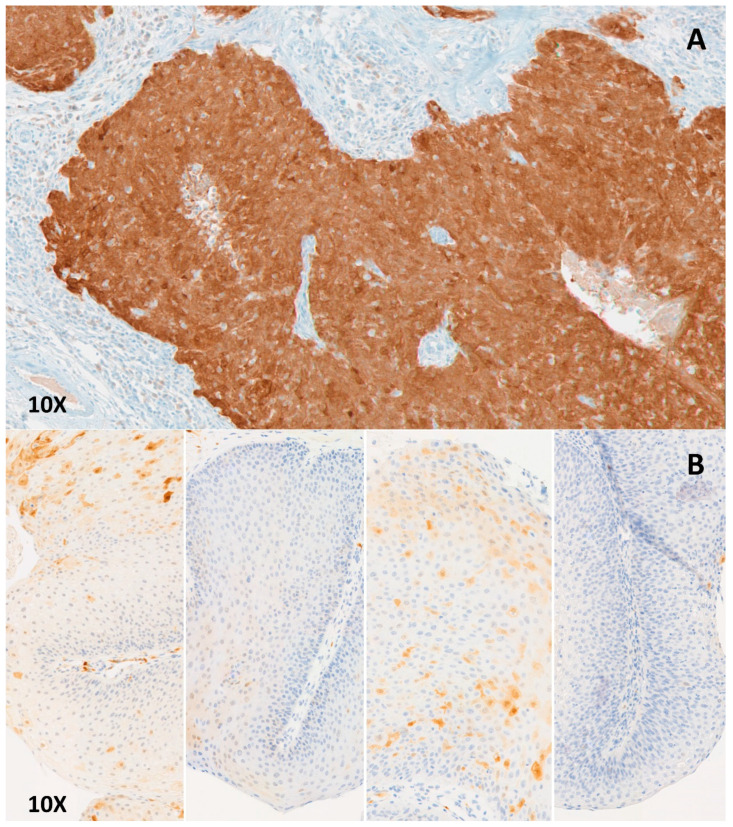
Examples of p16 immunostaining: (**A**) Positive immunostaining on an oropharyngeal squamous cell carcinoma related to HPV, nuclear and cytoplasmic staining of more than 70% of the tumoral cells. (**B**) Negative immunostaining: Papilloma in JoRRP from four patients, heterogenous nuclear and cytoplasmic staining of less than 70% of tumoral cells.

We conducted immunochemistry on FFPE sections of papilloma with p16^INK4a^ antibody (E6H4 clone, manufacturer: Roche, dilution: 1/2), with a Leica™ Bond III^®^ automat (Leica Microsystemes SA, Nanterre, France). The anti-p16^INK4a^ immunohistochemistry was rated positive when more than 70% of the cells displayed clear and homogeneous labeling of the cytoplasm and the nucleus, as shown in the Figure 2.

### 4.4. Statistical Analysis

Statistical analyses were carried out using the R software. Qualitative variables were analyzed with a Chi2 or Fisher test depending on the sample size. Univariate analyses with quantitative data were performed using a nonparametric Mann-Whitney test. Finally, all tests were bilateral and a *p* < 0.05 was considered significant.

### 4.5. Outcome

Patients were classified into two groups regarding their CISH score: score 1+ and score 2+. The primary outcome was the comparison of clinical markers of disease aggressiveness within these two groups. Secondary outcome was to explore the p16 expression in JoRRP’s lesions to find a correlation with a clinical outcome.

## 5. Conclusions

To conclude, we presented here the first results ever described in the JoRRP with a RNA CISH with a probe targeting E6 and E7 from HPV6 and 11. Patients with a high level of E6 and E7 transcription (score 2+) had a more aggressive disease compared to those with a low level of transcription (score 1+). These data are a first step towards the use of biomarkers predictive of disease severity. The use of biomarkers to predict an aggressive disease could improve the management of the disease, for example, by implementing adjuvant treatment at the early stages. It could also be an opportunity to better inform patients and their parents about the potential course of the disease. Our results also point to the potential of HPV6/11 RNA CISH as a predictive test of response to Cidofovir. These data require validation on a larger prospective cohort. 

## Figures and Tables

**Table 1:** Characteristics of the score 1+ and score 2+ populations.

Clinical Characteristics		Score 1+ (*n* = 19)	Score 2+ (*n* = 29)	*p*
Gender	Boys	10 (53%)	12 (41%)	0.444
	Girls	9 (47%)	17 (59%)	0.444
HPV typing ^1^	HPV6 and 11	1 (5%)	2 (7%)	1
	HPV11	5 (26%)	8 (27%)	0.923
	HPV6	12 (63%)	19 (66%)	0.867
Onset of disease at age ≤ 3 years		12 (63%)	14 (48%)	0.312
Tracheostomy		2 (10%)	6 (21%)	0.451
At least 1 Cidofovir injection		11 (57%)	24 (82%)	0.058
Extra-laryngeal involvement		5 (26%)	8 (27%)	0.923
Sub-glottic involvement		12 (63%)	21 (72%)	0.499
Tracheal involvement		5 (26%)	7 (24%)	0.8655
Postoperative morbidity		4 (21%)	6 (21%)	1
Lesion at last check-up		11 (63%)	23 (79%)	0.110
Pulmonary involvement		1 (5%)	3 (10%)	1
Death		0	1 (3%)	1
Malignant transformation		0	1 (3%)	1

^1^ one patient couldn’t have HPV typing due to sample depletion.

**Table 2:** Comparison of clinical markers of aggressiveness between score 1+ and 2+ populations; results are medians.

Clinical Markers of Aggressiveness	Score 1+ (*n* = 19)	Score 2+ (*n* = 29)	*p*
Age at diagnosis (year)	2	4	0.676
Years between 1st and last endoscopy	2.6	1.5	0.292
Number of SE per year	2.8	6.1	0.036
Total number of SE	8	9	0.128
Number of SE first year after diagnosis	2	5	0.029
Number of patient with more than 4 SE in one year	7	21	0.015
Average interval between each SE (days)	168	80	0.067
Number of Cidofovir injections	1	5	0.053
Cidofovir injection prior to the specimen	2	4	1
